# A comparison of photographic, replication and direct clinical examination methods for detecting developmental defects of enamel

**DOI:** 10.1186/1472-6831-11-16

**Published:** 2011-04-21

**Authors:** Ali Golkari, Aira Sabokseir, Hamid-Reza Pakshir, M Christopher Dean, Aubrey Sheiham, Richard G Watt

**Affiliations:** 1 Dept. of Epidemiology and Public Health, University College London, UK; 2 Dental School, Shiraz University of Medical Sciences, Iran; 3 Orthodontic Research Centre, Shiraz University of Medical Sciences, Iran; 4 Dept. of Cell and Developmental Biology, University College London, UK

## Abstract

**Background:**

Different methods have been used for detecting developmental defects of enamel (DDE). This study aimed to compare photographic and replication methods with the direct clinical examination method for detecting DDE in children's permanent incisors.

**Methods:**

110 8-10-year-old schoolchildren were randomly selected from an examined sample of 335 primary Shiraz school children. Modified DDE index was used in all three methods. Direct examinations were conducted by two calibrated examiners using flat oral mirrors and tongue blades. Photographs were taken using a digital SLR camera (Nikon D-80), macro lens, macro flashes, and matt flash filters. Impressions were taken using additional-curing silicon material and casts made in orthodontic stone. Impressions and models were both assessed using dental loupes (magnification=x3.5). Each photograph/impression/cast was assessed by two calibrated examiners. Reliability of methods was assessed using kappa agreement tests. Kappa agreement, McNemar's and two-sample proportion tests were used to compare results obtained by the photographic and replication methods with those obtained by the direct examination method.

**Results:**

Of the 110 invited children, 90 were photographed and 73 had impressions taken. The photographic method had higher reliability levels than the other two methods, and compared to the direct clinical examination detected significantly more subjects with DDE (P = 0.002), 3.1 times more DDE (P < 0.001) and 6.6 times more hypoplastic DDE (P < 0.001). The number of subjects with hypoplastic DDE detected by the replication method was not significantly higher than that detected by direct clinical examination (P = 0.166), but the replication detected 2.3 times more hypoplastic DDE lesions than the direct examination (P < 0.001).

**Conclusion:**

The photographic method was much more sensitive than direct clinical examination in detecting DDE and was the best of the three methods for epidemiological studies. The replication method provided less information about DDE compared to photography. Results of this study have implications for both epidemiological and detailed clinical studies on DDE.

## Background

Developmental defects of enamel (DDE) can be detected and studied using microscopic and macroscopic methods. Macroscopic methods are especially important in epidemiological studies. Direct clinical examination is the most widely used method for detecting enamel defects, while photographic and replication methods are of special interest because of their suggested advantages over direct clinical examination. None of these methods are fully standardized as no single detailed method is used by many researchers. The replication method used by some dental anatomists, archaeologists and anthropologists is not used by epidemiologists. The significance of digital technologies, which have opened up new horizons in almost all aspects of science, has been relatively neglected in epidemiological studies of DDE. Digital photography, which has been shown to have high levels of success in caries detection [1], has only been used in a few DDE studies.

Direct clinical examination is fast and cheap and all surfaces of teeth can be examined. However, it has many disadvantages such as observer bias and effects of visual problems related to fatigue of the examiner. Accuracy is highly dependent on cooperation of subjects [2,3]. Direct examination was unreliable when multiple indices were used or compared [4]. Direct visual examination of enamel can be done with or without tactile examination of the enamel surface with a probe [5]. Examination may be conducted under natural light, avoiding direct sunshine. When natural light is not strong enough or when posterior teeth are being examined, a fibre optic light may be used [6]. Teeth may be cleaned before the examination [7,8]. Polarizing filters may be used to overcome the burn outs from strong flashlight and to enhance the visual details of enamel defects, especially when the extent of defects is more important than their colour [9].

Photography has been used in some studies of tooth and enamel defects [3,4,9-14]. Assessing photographs is more objective than direct clinical examination. With photography it is possible for all cases (even from different geographic areas or examined at different times) to be assessed under standard conditions by one person or one group of examiners [11]. Photography facilitates randomization and blinding, so observer bias can be avoided. Photographs can be kept for future reassessment or application of different approaches or indices [3,12]. On the other hand, the disadvantages of photography are cost, technical sensitivity and inability to use tactility. Furthermore, with single photographs only labial surfaces of incisors are recorded. Multiple views are needed to view more teeth and/or more surfaces [3]. Some surfaces or parts of a surface may be missed even in multiple views. Some researchers have preferred to use conventional photography with 35 mm film [3,4,13]. However, digital photography provides better conditions to record developmental defects of enamel. Digital photography is cheaper and independent of developing negatives and printing or projection. Most importantly, it gives the photographer the opportunity to view each image immediately and repeat it in case there is any problem with the image, such as a burn-out caused by flash; or to take several photos and choose the best one later [15].

Replicas of teeth may be used in both macroscopic and microscopic studies of enamel defects. In this method the whole cast is in one colour, so changes in colour of enamel are not shown. But a replica of teeth gives the observer better visibility to investigate hypoplasia, including small changes in the enamel surface [16]. This method also enables researchers to spend as much time as needed and provides a dry specimen that can be studied easily from different perspectives without worrying about adjacent structures. Disadvantages of the replication method are cost, time needed to make replicas, and its sensitivity to technical methods. Even under the best conditions some proximal surfaces may not be well recorded. And, as stated above, it only displays hypoplastic defects.

As the three methods differ in sensitivity in detecting DDE, it is surprising that very few studies have compared them [3,4,14]. No published epidemiological study has compared the results of detecting DDE using the replication method with the direct examination on a population basis. Wong et al. (2005) used the Modified DDE Index to compare the photographic and direct examination methods and found kappa agreement values from 0.79 to 0.85 between them for detecting subjects with any DDE. They used one-view, three-view and five-view photographic methods. The highest prevalence of subjects with DDE (36.6%) was, surprisingly, obtained from their one-view method. It was close to the prevalence obtained by the direct clinical method (33.9%). The intra-examiner reliability of the photographic method (k = 0.81 to 0.88) was also close to the direct examination method (k = 0.82) [3]. Ellwood et al. (1996) used the TF (Thylstrup and Fejerskov) Index [16] for their comparison and found a substantial agreement between the two methods at subject level (k = 0.63). At subject level, the prevalence obtained by photographic method (44.9%) was close to that obtained by the direct examination (41.4%) [14]. Sabieha and Rock (1998) used both the Modified DDE Index and TF Index and reported almost perfect agreement between the direct examination and the photographic methods for both indices (k = 0.91 and 0.83 respectively). They only assessed maxillary central incisors [4].

Several clinical indices have been developed to categorize enamel defects based on their nature, appearance, microscopic features or their cause. Some indices, such as the TF Index [17], were introduced specifically for fluorosis. Other indices are descriptive and include all kinds of enamel defects including fluorosis. The Modified DDE Index [6] is a descriptive index derived from the original Developmental Defects of Enamel Index [18]. It covers all defects based on their macroscopic appearance. However, the criteria for classification are closely related with histo-pathological changes [19]. The Modified DDE index was claimed to be a more practical and comparable index in epidemiological studies. Its extensive use and its high degree of validity and reliability support that claim [6,20-22].

As there are few epidemiological studies comparing the three methods of detecting DDE, the objective of this study was to compare the ability of the digital photographic and replication methods with the direct clinical examination method to detect DDE in children's permanent incisors.

## Methods

The study was conducted on relatively newly erupted permanent incisors in a sample of 8 to 10-year-old school children of the city of Shiraz in the south of Iran. Approval and ethical permission were obtained from Iran's National Ethical Committee in Medical Research and the regional Educational Head Office. A representative sample of 335 primary schools children in grades 3 to 5 were first examined using the direct clinical examination method. Children were excluded from the study if they were outside the age range, had one or more permanent incisors unerupted or partially erupted, or had a fracture or restoration in their permanent incisors. Fifty five children with DDE and another 55 children without DDE were then randomly selected and invited to dental clinics for further investigation using the photographic and replication methods.

Upon arrival at the clinic the purpose and stages involved in the study were explained in detail to the parents. Parental consent was requested for taking photographs and impressions. DDE of permanent incisors were recorded based on the Modified DDE Index [6] in all three methods. Results of assessing photographs and replicas were separately compared with the results obtained from the direct examination.

### Direct clinical examination method

Direct intra-oral clinical examinations were carried out by 2 calibrated examiners in classrooms using natural light, disposable mirrors and tongue blades. Teeth were examined wet, but excess saliva and food debris was removed with sterilized gauze when necessary. Each of the two examiners checked the other examiner's findings on 1 in 10 random selected children to test the inter-examiner reliability. Testing the intra-examiner reliability was only possible if subjects were assessed again under the same conditions in schools. Permission for school re-visits was not granted.

### The Photographic method

A Nikon D80 digital SLR (Single Lens Reflex) camera with a 105 mm macro lens which provided a magnification of 1:1 and a macro double flash was used. The quality of photos was set on "JPEG FINE" and *5.2 *mega pixels. Speed and diaphragm were set on 60 and 32 based on the best results obtained from a pilot study of 13 children chosen from the same population.

Photographs were taken by two calibrated photographers with the child sitting on a dental chair and leaning back to avoid movement during focusing and taking photographs. Cheeks and lips were retracted so that all 12 anterior teeth and part of the upper and lower gums were shown. An assistant helped with retraction of cheeks in less-cooperative subjects. The child was asked to close the incisors together edge to edge. This was practiced a few times while holding a mirror in front of their face before taking the photograph. Food debris and/or excess saliva was removed with sterilized gauze when necessary. A "one view" photograph was considered to be acceptable as only incisors were assessed [3]. Photographs were taken by focusing on the centre of the 4 central incisors. The camera was planed approximately 15 degrees above the perpendicular to the central incisors' plane to minimize specula reflection and burn outs. Each photograph was evaluated for acceptability and quality. If not acceptable, the photograph was repeated.

Photographs were viewed randomly on a 17" flat free-angle monitor with high resolution (1028 by 1024 pixels) using "Adobe Photoshop CS version 8.0" software. Each photograph was assessed and scored independently by two calibrated examiners. Each photograph was first viewed as actual size based on the magnification ratio of 1:1 used when taking them. Then the examiner could use the magnifier tool to enlarge the photo several times. Some defects were clearer when using higher magnifications but some others were better seen with lower magnifications when the sharpness of components was higher.

The examiners' first records were used to assess inter-examiner reliability. Disagreements between examiners on coding a DDE were then solved by reassessment of teeth by both examiners for the purpose of comparing the photographic and direct examination methods. To test the intra-examiner reliability in the photographic method, each of the two examiners re-rated all photographs in a computer generated random order a year after the original assessment.

### The Replication method

Impressions were taken of the anterior teeth of children using heavy body (putty) and then light body (liner) of Affinis, an additional curing rubber base material made by Coltène/Whaledent. The impressions were disinfected by soaking in sodium hypochlorite for five minutes and stored in individual bags away from heat, direct sunshine and pressure. They were cast using hard orthodontic stone. All casts were assessed for hypoplastic defects by two calibrated examiners. All casts and impressions were viewed using a pair of dental loupes with a magnification of x3.5. Type of DDE detected in replicas and impressions were also recorded using the Modified DDE Index and its subcategories, as mentioned above. However, considering the fact that opacities were not detectable in this method, codes given to detected DDE were limited to 7 (pits) and 8 (missing enamel).

Similar to the photographic assessment, examiners' first records were used to test the inter-examiner reliability in replication assessment and then disagreements were resolved by discussion. However, in this method impressions and casts were both reassessed and compared. To test the intra-examiner reliability, casts and impressions were reassessed by the two examiners a year after the original scoring. Unlike the photographs, avoiding bias by recognition of impressions and casts was not possible. This occurred because replications were not the same shape or colour and lacked a randomised environment such as that done by computer in the photographic reassessment. However, the time period between the first assessment and the reassessment (one year) was long enough to assume it was not possible for examiners to remember their first scoring at the time of the reassessment.

### Comparison of methods

The number and percentage of children with DDE and teeth with DDE detected by each method was calculated. The total number of DDE detected by each method was also calculated as several defects may be present on one tooth. Such calculations made it possible to compare the results obtained by photographic and replication methods with those of direct examination method at individual, tooth, and lesion levels. All types of DDE were included in comparison of direct examination and photographic methods. However, only hypoplastic defects were taken into account when comparing the direct examination and replication methods as colour changes were not recorded in the replicas. Kappa agreement was used to test reliability of methods and the agreement between each of the two photographic and replication methods and the direct examination method. Two-sample proportion test was used to evaluate if the prevalences obtained by the two methods were significantly different. McNemar's test was used to test whether number of subjects or teeth with DDE detected only by one method was significantly higher than the other method. The data collection took place in Spring 2007. The reassessment of photographs and models was done in Spring 2008. SPSS (Version 14) software was used for analysis.

## Results

The number of children examined at schools, invited to clinics, had their photographs taken, and had impressions taken of their teeth are shown in Table 1. Ninety five of the invited 110 children attended the clinics. However, some parents did not give consent for photographs or impressions to be taken. Photographs were taken of 90 children (81.8% response rate). Therefore, the direct examination and photographic methods were compared based on these 90 children. Impressions were taken of teeth of 73 children (66.4% response rate) which were used for comparison of the replication and direct examination methods.

**Table 1:** Number of children in each part of study, by sex.

Sex	Were examined at schools	Were invited to clinics	Had photographs taken	Had impressions taken
Girls	177	56	48	42
Boys	158	54	42	31

Total	335	110	90	73

Results of the inter-examiner and intra-examiner reliability tests for each test are shown in Table 2. As explained earlier, intra-examiner reliability was not tested for the direct clinical method. The intra-examiner reliability for photographic and replication methods are the average of the two examiners. 14 disagreements were found between the two examiners in assessing the photographs. 10 of them (72%) occurred when one of the examiners missed detecting or reporting a DDE on a tooth with 2 or more defects. 2 disagreements (14%) occurred when a diffuse opacity was coded as "lines" by one examiner and as "patchy" by the other one. Two other disagreements (14%) were based on colour of demarcated opacities. Eight disagreements were found in replication assessments, all being related to distinguishing a DDE from an artefact.

**Table 2:** Inter-examiner and average intra-examiner reliability levels of each method in detecting DDE.

Type of DDE	Method	Sample size	Inter-examiner reliability			Intra-examiner reliability		
			
			Individual level	Tooth level	Lesion level	Individual level	Tooth level	Lesion level
All types of DDE	Direct examination	38	0.90	0.84	0.81	-	-	-
	Photographic	90	1.00	1.00	0.90	1.00	0.99	0.93
	Replication	-	-	-	-	-	-	-

Hypoplastic DDE	Direct examination	38	1.00	0.91	0.91	-	-	-
	Photographic	90	1.00	1.00	0.95	1.00	0.99	0.95
	Replication	73	0.93	0.80	0.78	0.94	0.84	0.80

The photographic method detected all cases with DDE that were detected clinically, except one. Twenty cases were only detected by the photographic method. The photographic method detected 1.4 times more children with DDE, 2.1 times more teeth with DDE, and 3.1 times more DDE lesions than the direct examination method. The photographic method also detected 4.5 times more individuals with hypoplastic DDE, 5.9 times more teeth with hypoplastic DDE, and 6.6 times more hypoplastic lesions than the direct examination method (Table 3). There was a moderate agreement (k = 0.48) between the two methods in detecting cases with DDE at subject level. The photographic method detected significantly more DDE than the direct examination method for the following: number of subjects with DDE (P = 0.002), number of teeth with DDE (P < 0.001), number of subjects with hypoplastic defects (P < 0.001), and number of teeth with hypoplastic defects (P < 0.001). In addition to detecting higher numbers than the direct examination, the numbers detected only by the photographic method were significantly higher than the numbers detected only by the direct examination for all 4 tests (P < 0.001).

**Table 3:** Comparison of direct examination and photographic methods in detecting all types of DDE and in detecting hypoplastic DDE in permanent incisors of 90 children.

Type of DDE	Method	Number and percent of subjects with DDE (%)	Number and percent of teeth with DDE (% of all examined teeth)	Mean number of teeth with DDE per child in all 90 children	Mean number of teeth with DDE per affected child	*Number of DDE in all 90 children	*Mean DDE per child in all 90 children	*Mean DDE per affected child	*Mean DDE per affected tooth
All types of DDE	Direct examination	50 (55.6)	115 (16.0)	1.3	2.3	121	1.3	2.4	1.1
	Photographic method	69 (76.7)	246 (34.2)	2.7	3.6	374	4.2	5.4	1.5

Hypoplastic DDE	Direct examination	11 (12.2)	20 (2.8)	0.2	1.8	24	0.3	2.2	1.2
	Photographic method	49 (54.4)	117 (16.3)	1.3	2.4	159	1.8	3.3	1.4

*Note: Some teeth had more than one DDE.

One hundred and one teeth (88% of those detected by direct examination and 41% of those detected by photographic method) had DDE detected by both direct examination and photographic methods. Fifty four teeth (47% of those detected by direct examination and 22% of those detected by photography) were identically scored by both methods. The number increased from 54 to 66 teeth when similar subcategories, scores 1 and 2, 3 to 5, and 7 and 8, were combined. Scores 6 and 9 were not combined with any other subcategory. At the lesion level, 56 DDE lesions (46% of those detected by direct examination and 15% of those detected by photographic method) were identically scored by both methods. The number increased to 70 defects when similar subcategories were combined. Diffuse opacities had the highest prevalence in both methods.

In the 73 children who had impressions taken of their teeth, the replication method detected 1.5 times more children with hypoplastic DDE, 2.4 times more teeth with hypoplastic DDE, and 2.3 times more hypoplastic DDE lesions than the direct clinical examination method (Table 4). 21 hypoplastic defects (87.5% of those detected by direct clinical examination and 37.5% of those detected by the replication method) were detected by both methods. The prevalence of subjects with hypoplastic DDE was not significantly different between the replication and the direct clinical examination methods (P = 0.166). However, the proportion of teeth with hypoplastic DDE detected by the replication method was significantly higher than that obtained by direct examination method (P < 0.001). On the other hand, the number of affected subjects and teeth detected only by the replication method were significantly higher than those detected only by the direct clinical examination method (P < 0.001).

**Table 4:** Comparison of direct examination and replication methods in detecting hypoplastic DDE in permanent incisors of 73 children.

Method	Number and percent of subjects with DDE (%)	Number and percent of teeth with DDE (% of all examined teeth)	Mean number of teeth with DDE per child in all 73 children	Mean number of teeth with DDE per affected child	*Number of DDE in all 73 children	*Mean DDE per child in all 73 children	*Mean DDE per affected child	*Mean DDE per affected tooth
Direct examination	13 (17.8)	20 (3.4)	0.3	1.5	24	0.3	1.9	1.2
Replication method	20 (27.4)	47 (8.1)	0.6	2.35	56	0.8	2.8	1.2

*Note: some teeth had more than one DDE in them.

The average time spent on direct clinical examination of each child was about 3 minutes. Taking photographs of each child, including the time spent for preparing the child and repeating the photograph (if necessary) took less than one minute. Taking impressions took 15 minutes on average. Examiners were told to spend as much as time as they needed to assess the photographs and replicas. Assessing a photograph took 6.5 minutes in average. Assessing replicas took up to 20, and in average, 12 minutes.

## Discussion

It was assumed that the photographic method would detect more changes in colour and transparency of enamel than the direct clinical examination method. But the ability of the photographic method to detect hypoplastic defects, where defective enamel had the same colour as its surroundings, was in doubt. The replication method, on the other hand, although not showing changes in colour, was assumed to better detect the hypoplastic DDE than the other two methods as it was the usual method used by dental anthropologists and anatomists to find fine hypoplasias. Results of this study, however, suggest that the photographic method was good enough to detect both hypomineralized and hypoplastic enamel defects. The photographic method, whilst not necessarily the most sensitive method, detected considerably more DDE of all types, 3.1 times more DDE lesions in total, than the direct examination method.

This study found only a moderate agreement (k = 0.48) between the direct clinical examination and photographic methods in detecting DDE at subject level. These findings differ from other epidemiological studies comparing the photographic and direct clinical examination methods. They reported kappa values of 0.63 [14] to 0.91 [4]. The low level of agreement found in this study was not due to the inability of the photographic method to detect DDE, but due to significantly more subjects with DDE being detected by the photographic method than the direct examination method (P = 0.002). Results of the study by Ellwood et al. [14] also showed significant differences between the numbers of subjects with DDE detected by the two methods, although they found a much smaller difference. The other two studies mentioned above did not find such results [3,4]. Indeed they reported very similar prevalence of cases with DDE detected by the two methods. Wong et al. [3] reported that 33.9% of their subjects had a DDE detected by the direct examination with a very similar percentage of 34.6% to 36.6% detected by the photographic method. The photographic method used in the present study was able to detect most (98%) of the cases detected by the direct examination method plus a significant number of new cases. None of the three above-mentioned studies reported a similar finding.

As with the comparisons at subject level, at the tooth level the photographic method detected most of the DDE detected by direct examination plus significantly more other affected teeth. Comparing the results of the present study with those of Sabieha and Rock [4] in detecting a DDE in permanent upper central incisors, both studies found that the photographic method detected around 82% of those upper central incisors detected by the direct examination (46 out of 56 in this study, and 161 out of 194 in the study by Sabieha and Rock). However, the percentage of affected upper central incisors detected only by the photographic method in the present study (27%) was three times greater than the 9% reported by Sabieha and Rock [4] (P < 0.001).

These findings show that the photographic method used in this study detected more DDE than both the direct clinical examination method used in this study and the photographic methods used in other studies at both subject and tooth level. Unfortunately no previous study compared the two methods at lesion level. The main differences in the methods used in this study from those used in the above-mentioned studies are that in the present study a powerful digital camera with well tested accessories and settings was used. That allowed the photographer to zoom and focus to have the best picture of the 8 incisors instead of using a fixed barrel lens, and allowed the examiners to view the photographs at different magnifications and angles, as they were able to do during the direct clinical examination.

Both photographic and replication methods provided permanent records of teeth, but the photographic method also provided for easy random presentation of subjects with less bias than in the other methods. The photographic method was also faster than replicas, both in time needed to be taken (1 versus 15 minutes) and in time to be assessed (6.5 versus 12 minutes), with no laboratory process and no concerns about cross infection. A clinical setting or presence of a dental clinician was not necessary for taking photographs. Unlike the photographic method, the replication method showed lower inter-examiner reliability levels than the direct clinical examination method (Table 2).

## Conclusion

The digital photographic method detected much more DDE than the direct examination method and provided much more information than the replication method. Therefore, the digital photographic method, as used in this study, was the best of three methods used for detecting enamel defects of permanent incisors of children. Results of this study have implications for both epidemiological and detailed clinical studies on DDE.

## Competing interests

The authors declare that they have no competing interests.

## Authors' contributions

This paper is based on a PhD thesis by AG done under the supervision of RGW and ASh. ASa helped with data collection, processing and analysis. The fieldwork was planned and supervised by HRP. MCD helped with the design of the study and provided technical advice and support. AG wrote the paper. All authors read and approved the final manuscript.

## Pre-publication history

The pre-publication history for this paper can be accessed here:

http://www.biomedcentral.com/1472-6831/11/16/prepub
